# Ulceration of the Bladder: Simple, Tuberculous, and Malignant

**Published:** 1901-03

**Authors:** 


**Ulceration of the Bladder: Simple, Tuberculous, and Malignant.** By E. Hurry Fenwick. Pp. vi., 85. London: J. & A. Churchill. 1900.

The subject dealt with in these pages is a difficult and obscure one. The author's ten years' incessant practice with the cystoscope, and his records of observations on cases, many of which have been watched for long periods—some for ten or fifteen years, place him in a position to speak with special authority on the types of disease which are considered. After a preliminary consideration of the difficulties and dangers which attend instrumentation, especially cystoscopy, in most cases of bladder ulceration, a form of simple ulcer is described comparable with that which affects the stomach. The characters of the ulcer, its history, and symptoms, are given. The difficulty in effecting a diagnosis between this ulcer and a solitary tuberculous ulcer is adequately recognised. Visual aspects do not enable the distinction to be made; the course of the cases must be watched for long periods, and evidence of the presence or absence of other possible tubercular lesions in the genito-urinary organs carefully searched for. The various forms of tubercular disease and ulceration of the bladder are next considered under the headings of primary tuberculosis of the organ, and tubercular affections secondary to primary tuberculosis of the epididymis, prostate, or kidney. Finally, malignant ulceration is considered, the author reserving this term for that form of epithelioma of the bladder which is visually comparable to rodent or epitheliomatous ulcer of the skin. The treatment recommended for these various forms of ulceration in their different stages is clearly described.

We regard the book as a very valuable and suggestive record of observations and opinions upon the subject with which it deals. Future researches of the author, and of others engaged in similar studies, may be expected to corroborate or modify the many original opinions which are advanced — all of which opinions deserve serious attention and criticism, being obviously the outcome of long and laborious investigations.

